# ANOCRITICAL READINGS OF THE INTERSECTIONS OF AGE AND GENDER IN AMERICAN LITERATURE: A FEMINIST CRITIQUE

**DOI:** 10.1093/geroni/igac059.1489

**Published:** 2022-12-20

**Authors:** Nicole Haring

**Affiliations:** University of Graz, Graz, Steiermark, Austria

## Abstract

Intergenerational relationships have been present features in texts from the American literary canon that often problematizes the intersections of age and gender. This paper investigates how representations of intergenerationality provide the opportunity to explore the concepts of relationality and intersectionality through a feminist lens. Maierhofer’s concept of anocriticism was used in in the analysis of Julia Alvarez’ In the Time of the Butterflies (1994) and Yaa Gyasi’s novel Homegoing (2016) to validate individual experiences of gendered ageing. This paper presents ways in which this particular intersection can be viewed as a potential site of resistance towards what it means to grow old as a woman. Placing literature in a cultural, social, and political context, traditional paradigms can be reconstructed and heteronormative assumptions of age and gender can be deconstructed by focusing on the individual narratives and their potential for resistance.

